# Deciphering the immunological and prognostic features of bladder cancer through platinum-resistance-related genes analysis and identifying potential therapeutic target P4HB

**DOI:** 10.3389/fimmu.2023.1253586

**Published:** 2023-09-18

**Authors:** Situ Xiong, Sheng Li, Jin Zeng, Jianqiang Nie, Taobin Liu, Xiaoqiang Liu, Luyao Chen, Bin Fu, Jun Deng, Songhui Xu

**Affiliations:** ^1^ Department of Urology, The First Affiliated Hospital of Nanchang University, Nanchang, China; ^2^ Jiangxi Institute of Urology, Nanchang, China

**Keywords:** platinum resistance, bladder cancer, molecular subtypes, immune microenvironment, immunotherapy, prognosis, P4HB

## Abstract

**Objectives:**

To identify the molecular subtypes and develop a scoring system for the tumor immune microenvironment (TIME) and prognostic features of bladder cancer (BLCA) based on the platinum-resistance-related (PRR) genes analysis while identifying P4HB as a potential therapeutic target.

**Methods:**

In this study, we analyzed gene expression data and clinical information of 594 BLCA samples. We used unsupervised clustering to identify molecular subtypes based on the expression levels of PRR genes. Functional and pathway enrichment analyses were performed to understand the biological activities of these subtypes. We also assessed the TIME and developed a prognostic signature and scoring system. Moreover, we analyzed the efficacy of immune checkpoint inhibitors. Then we conducted real-time fluorescence quantitative polymerase chain reaction (RT-qPCR) experiments to detect the expression level of prolyl 4-hydroxylase subunit beta (P4HB) in BLCA cell lines. Transfection of small interference ribonucleic acid (siRNA) was performed in 5637 and EJ cells to knock down P4HB, and the impact of P4HB on cellular functions was evaluated through wound-healing and transwell assays. Finally, siRNA transfection of P4HB was performed in the cisplatin-resistant T24 cell to assess its impact on the sensitivity of BLCA to platinum-based chemotherapy drugs.

**Results:**

In a cohort of 594 BLCA samples (TCGA-BLCA, n=406; GSE13507, n=188), 846 PRR-associated genes were identified by intersecting BLCA expression data from TCGA and GEO databases with the PRR genes from the HGSOC-Platinum database. Univariate Cox regression analysis revealed 264 PRR genes linked to BLCA prognosis. We identified three molecular subtypes (Cluster A-C) and the PRR scoring system based on PRR genes. Cluster C exhibited a better prognosis and lower immune cell infiltration compared to the other Clusters A and B. The high PRR score group was significantly associated with an immunosuppressive tumor microenvironment, poor clinical-pathological features, and a poor prognosis. Furthermore, the high PRR group showed higher expression of immune checkpoint molecules and a poorer response to immune checkpoint inhibitors than the low PRR group. The key PRR gene P4HB was highly expressed in BLCA cell lines, and cellular functional experiments *in vitro* indicate that P4HB may be an important factor influencing BLCA migration and invasion.

**Conclusion:**

Our study demonstrates that the PRR signatures are significantly associated with clinical-pathological features, the TIME, and prognostic features. The key PRR gene, P4HB, s a biomarker for the individualized treatment of BLCA patients.

## 1 Introduction

Bladder cancer (BLCA) is a prevalent malignancy of the urinary system, with an estimated 573,000 new cases and 212,000 deaths occurring annually worldwide (1). Three-quarters of newly diagnosed BLCAs are non-muscle invasive BLCAs (NMIBCs), which are usually treated by transurethral resection followed by intravesical chemotherapy or bacilli Calmette-Guerin (BCG) therapy (2). However, nearly 10 to 20% of NMIBC cases eventually progress to muscle-invasive BLCA (MIBC), an aggressive disease (3). Radical cystectomy plus pelvic node dissection remains the basic management for MIBC; however, the 5-year survival rate ranges from only 20% to 40%, and nearly 50% of cases progress to a metastatic state within three years (4).

Platinum-based chemotherapy has become a critical adjuvant therapy for metastatic BLCA patients since the late 1980s (5). However, drug resistance is a significant obstacle to successful treatment. Platinum-based chemotherapy is supported by level 1 evidence as the first-line management strategy against advanced urothelial cancer with relatively high objective response rates of 36-65% (6). Despite its objective efficacy, they fail to improve survival rates for metastatic BLCA patients, with only 13-15% achieving a 5-year overall survival rate (6). This is consistent with the development of chemoresistance seen in nearly all BLCA patients as the disease progresses, which obstacles to successful treatment and contributes to poor prognosis.

Tumor heterogeneity is a major challenge in oncology, as it is one of the hallmarks of cancer and a leading cause of drug resistance, ultimately resulting in therapeutic failure. The complex nature of tumor heterogeneity can influence drug resistance by directly affecting therapeutic targets or shaping the tumor microenvironment (TME) by defining unique transcriptomic and phenotypic profiles (7). As tumors evolve and progress, tumor heterogeneity can change spatially and temporally, leading to a constantly evolving TME that can challenge effective treatment strategies (7). Therefore, a better understanding of the TME and its role in platinum resistance could provide important insights into the resistance mechanisms and help identify new therapeutic targets for BLCA patients.

This study aims to investigate the role of the platinum-resistance-related (PRR) signatures in the tumor immune microenvironment (TIME) and prognostic features for BLCA patients through molecular subtypes and scoring system. By understanding the complex relationship between platinum resistance and the immune microenvironment in BLCA, we hope to provide new insights into the pathogenesis of platinum resistance and improve clinical management for BLCA patients at risk of platinum resistance.

## 2 Materials and methods

### 2.1 BLCA datasets and preprocessing

We collected open gene expression data with full annotation of clinical information of 687 samples from the public databases of The Cancer Genome Atlas (TCGA; n=431) database (https://portal.gdc.cancer.gov/) and Gene Expression Omnibus (GEO; n=256) database (GSE13507; https://www.ncbi.nlm.nih.gov/geo/). We obtained the PRR genes from the HGSOC-Platinum database (http://ptrc-ddr.cptac-data-view.org). We accessed The Cancer Immune Atlas (TCIA) database (https://www.tcia.at/home) to download the results of CIBERSORT using gene expression of the TCGA-BLCA dataset. We used the “limma” package of R software to construct a differential gene expression matrix for the PRR genes in BLCA patients.

### 2.2 Identification of PRR molecular subtypes

We took the intersection of the expressed genes of BLCA in the TCGA and GEO databases and the PRR genes in the HGSOC-Platinum database to obtain initial intersection genes. Then, the PRR genes were recognized by univariate Cox proportional hazards analysis based on the threshold of *p*<0.05. We performed unsupervised clustering analysis on BLCA samples based on the expression levels of these PRR genes. We used consensus clustering algorithms to determine the number of clusters of the samples. We used the “ConsensusClusterPlus” package in R to perform a cluster analysis on all samples. We applied the empirical Bayesian method in the “limma” R package to identify differentially expressed genes (DEGs) between the PRR clusters (*p*<0.05). Finally, the DEGs were used to perform cluster analysis to validate the accuracy of the clustering further. Principal component analysis (PCA) was performed with the “prcomp” function in the “stats” R package for dimensionality reduction. The results of PCA were visualized with the “ggbiplot” R package.

### 2.3 Assessment of TIME

Utilizing the CIBERSORT algorithm in R software, we analyze the RNA-Seq data of BLCA samples and the gene expression feature set of immune cell subtypes to determine the relative proportions of infiltrating immune cells for each BLCA patient. Then, we employed the ESTIMATE method in R to evaluate each sample’s immune and ESTIMATE scores. Furthermore, to identify the TIME cell infiltration, we used the single-sample Gene Set Enrichment Analysis (ssGSEA) algorithm to assess the enrichment of 28 immune cells in each patient.

### 2.4 Development of the PRR scoring system

The optimal PRR genes were subsequently determined by least absolute shrinkage and selection operator (LASSO)-penalized Cox regression. Then, these PRR genes with the best predictive value were entered into the multivariate Cox proportional hazards regression model. The PRR scoring system was developed based on a linear combination of the regression coefficients derived from the multivariate Cox proportional hazards regression model coefficients multiplied by normalized PRR gene expression levels. We divided the BLCA patients into the high PRR score group and the low PRR score based on the optimum cutoff value from the score obtained by the surv_cutpoint function of the “Survminer” R package. The Kaplan–Meier (K-M) survival analysis was performed between the groups through the R packages “survival” and “survminer”, and the results were visualized with K-M curves. The area under the curve (AUC) of the receiver operating characteristic (ROC) curve obtained by the “survival ROC” R package was used to estimate the predictive performance of the PRR scoring system.

### 2.5 Functional and pathway enrichment analysis

We applied “GSVA” R packages, a non-parametric and unsupervised method, to assess the enrichment of gene sets to ascertain the potential differences in biological functions among the high- and low-PRR score groups. The GSVA algorithm was performed to score each gene set comprehensively. The gene sets in our study were obtained from the Molecular Signatures Database. Additionally, we used the “ClusterProfiler” to get functional annotation of the PRR signatures based on Gene Ontology (GO) terms and Kyoto Encyclopedia of Genes and Genomes (KEGG) pathways. Differences were indicated statistically significant in GO when *p* values were less than 0.05.

### 2.6 Identification of cohorts with immune-checkpoint blockade

We downloaded survival, follow-up, and immunotherapy effect information of patients with metastatic urothelial cancer treated with prephenate dehydratase 1/programmed cell death 1 ligand 1 (PD1/PD-L1) from the IMvigor210 database (http://research-pub.gene.com/IMvigor210CoreBiologies). We exclude the patients without complete clinical information. We applied the DEseq2 R package to normalized raw count data.

### 2.7 Validation of prolyl 4-hydroxylase subunit beta (P4HB) expression in BLCA cell line

The expression levels of key PRR genes were verified using real-time fluorescence quantitative polymerase chain reaction (RT-qPCR). RNA extraction was performed using TRIzol reagent (Qiagen, USA), followed by reverse transcription using the Takara PrimeScript RT kit (Qiagen, USA). Subsequently, RT-qPCR was conducted on the Roche LightCycler96 RT-qPCR system using the SYBR premix Ex Taq kit (Takara Bio, Inc., Otsu, Japan). The FC in messenger ribonucleic acid (mRNA) expression was calculated using the 2^−ΔΔCt method. Primer sequences:

P4HB_F: TCACCAAGGAGAACCTACTGGA.

P4HB_R: GGCAAGAACAGCAGGATGTGAG.

### 2.8 Cell culture and small interference ribonucleic acid (siRNA) transfection

Human BLCA cell lines 5637 and EJ were obtained from the cell bank of Type Culture Collection of Chinese Academy of Science (Shanghai, China) and T24 were obtained from Procell Life Science&Technology Co., Ltd (Wuhan, China). Cells incubated at 37°C in a 5% CO2 humidified incubator. 5637 and EJ cells were cultivated in RPMI-1640 (Gibco, USA), and T24 cell was cultured in Dulbecco’s modified eagle medium (DMEM) (Gibco, USA). All the media were replenished with 10% fetal bovine serum (FBS, Gibco, Australia) and 1% penicillin-streptomycin (New Cell Molecular Biotech Co., Ltd, China). Cells were plated into a 6-well plate at a density of 70–90%. siRNA of P4HB and its siRNA control were synthesized by RiboBio Co., Ltd. (Guangzhou, China) and then transiently transfected using Lipofectamine 2000 (Invitrogen) according to the manufacturer’s description. The sequences of siRNA sequences were P4HB siRNA-1 (5’-CCAUCAAGUUCUUCAGGAAUGTT-3’), P4HB siRNA-2 (5’- GUGACGUGUUCUCCAAAUACCTT-3’), and NC siRNA (5’-UUCUCCGAACGUGUCACGUTT-3’).

### 2.9 Cell migration by wound−healing and transwell assays

The wound healing and transwell assays were conducted to assess the migratory capacity of cells. Cells were seeded overnight at a density of 30% per well in 2 mL of medium in 6-well plates. Following siRNA transfection, a straight linear wound was created in each well using a 1 mL pipette tip. Subsequently, the cells were gently rinsed with PBS to eliminate cellular debris and cultured in DMEM supplemented with 5% FBS. Finally, wound healing images were captured at 0 and 24 hours using an inverted microscope (Olympus CKX41). 5637 and EJ cells were seeded at a density of 1×10^5 cells per well onto the upper chambers (8 μm pore size, Corning Inc., NY-Corning, USA) with 200 μL of serum-free medium, while the lower chambers were filled with 500 μL of medium containing 10% FBS. The cells were allowed to migrate for 24 hours. Subsequently, five random fields were selected for image capture, and the number of cells passing through the chamber was quantified.

### 2.10 Cell counting kit-8 (CCK8) assay

CCK-8 assay was performed to evaluate cell viability and proliferation. Cells (T24, PRR-T24, and siP4HB-PRR-T24) were seeded in 96-well plates at a density of 4000 cells per well and allowed to adhere overnight. The methodology for generating platinum-resistant T24 cells can be found in our previous study (8). The culture medium was replaced with a fresh medium containing CCK-8 reagent (Nanjing Keygen Biotech Co., Ltd., Nanjing, China). Following incubation for a 2h, the absorbance was measured using a microplate reader at a wavelength of 450 nm. The absorbance values of siP4HB-PRR-T24 were used to calculate the relative cell viability and proliferation compared to the control group (T24 and PRR-T24 groups).

### 2.11 Statistical analysis

A two-sided Wilcoxon rank-sum test was used to analyze the difference for continuous variables with the nonnormal distribution. The one-way ANOVA and Kruskal-Wallis tests were performed for differential analysis between the groups. Univariate and multivariate Cox proportional hazards regression analyses were used to determine the independent prognostic factors. Survival curves were visualized using the K–M method, and the Log-rank test evaluated the difference. Spearman’s correlation test assessed the correlations between continuous variables. In comparisons among groups, *p*<0.05 was indicated to be statistically significant differences. The R software (v4.2.2) was used to perform all statistical analyses.

## 3 Results

### 3.1 The genetic characteristics of the PRR signatures in BLCA

**Figure 1:**
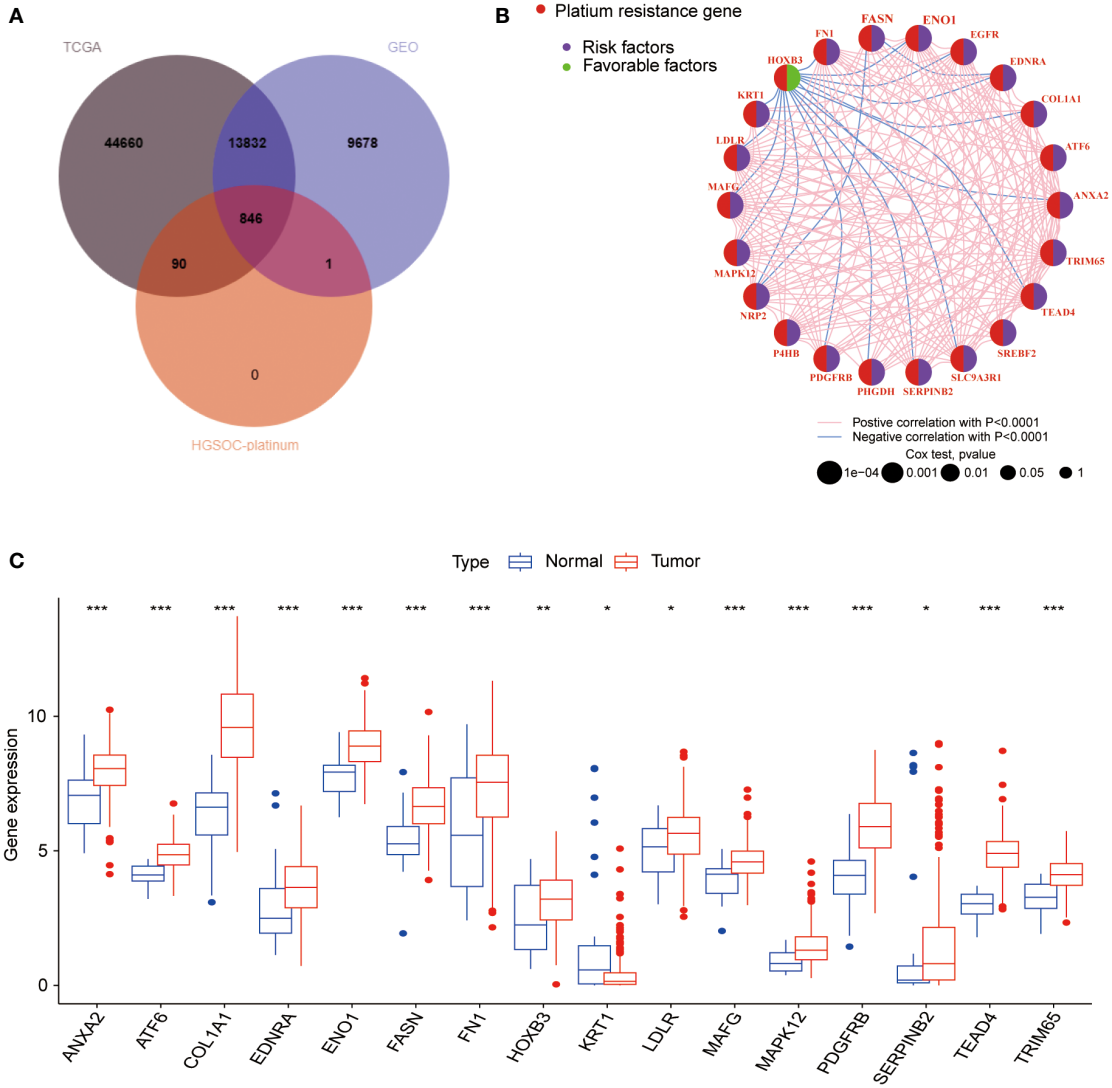
Analysis of the PRR gene expression and prognosis in BLCA. **(A)** The intersection genes between the TCGA, GEO databases and the PRR genes from the HGSOC-Platinum database. **(B)** The network of the interaction and regulatory relationships of the PRR genes with significant prognostic relevance. **(C)** Differential expression analysis of the prognosis-related PRR genes between normal and tumor tissues. (*p< 0.05; **p< 0.01; ***p< 0.001).

The overall design of the construction of the PRR patterns and the signature in BLCA was shown in **Figure S1**. In this study, we included 594 BLCA samples (TCGA-BLCA, n=406; GSE13507, n=188) with complete clinical and survival information (**Table S1**). We initially obtained 846 PRR genes associated with BLCA by intersecting the expression genes of BLCA in the TCGA and GEO databases with the PRR genes in the HGSOC-Platinum database (**Figure 1A**, **Table S2**). After univariate Cox proportional hazards regression analysis, 264 PRR genes were identified that were associated with prognosis in BLCA (*p*<0.05, **Table S3**). The network of the interaction and regulatory relationships of the PRR genes with significant prognostic relevance (n=22, *p*<0.001) is shown in **Figure 1B**, and the expression of their differentially expressed (n=16) between the normal and tumor tissue was visualized by box plot (**Figure 1C**).

### 3.2 Identification of the PRR molecular subtypes in BLCA

**Figure 2:**
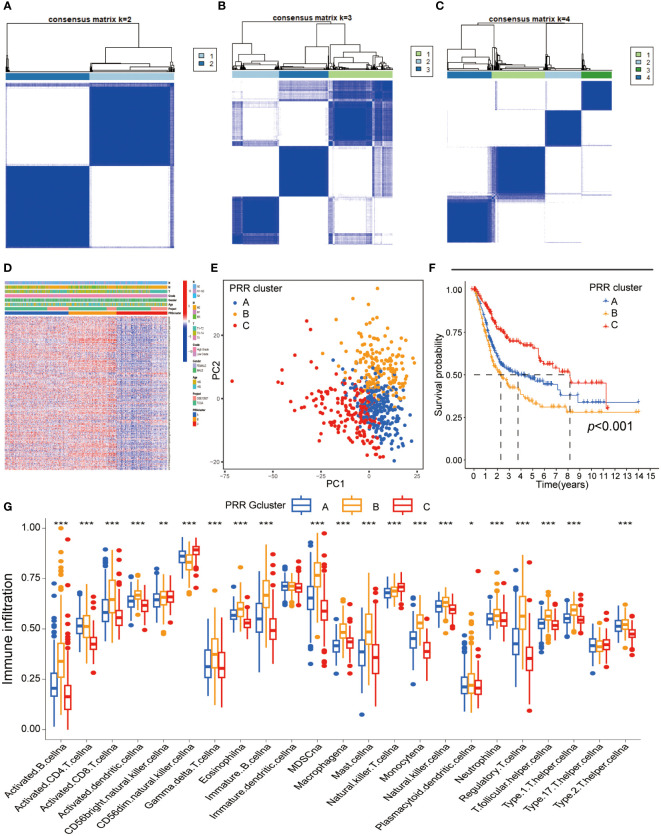
Identification of the PRR molecular subtypes in BLCA. **(A-C)** The consensus matrix heatmap demonstrated that the optimal clustering solution for consensus clustering was K=2 **(D)** The heatmap visualizing differential expression of the 264 PRR genes between three different molecular subtypes **(E)** Principal component analysis of the PRR genes identified three different molecular subtypes. **(F)** K-M curves for overall survival of the three PRR clusters in BLCA patients **(G)** Immune cell infiltration abundance of the three PRR molecular subtypes. (*p< 0.05; **p< 0.01; ***p< 0.001).

We performed the unsupervised clustering analysis of the expression of 264 PRR genes in 594 samples to explore the classification of BLCA by increasing the clustering matrix (k) from 2 to 4. As a result, consensus clustering was most suitable when k=3, and three molecular subtypes were identified and named Cluster A-C (**Figures 2A-C**). Cluster A contained 237 cases, Cluster B contained 174 cases, and Cluster C contained 183 cases. The relationship between the three molecular subtypes and the clinical features, including the TNN stage, grade, and age, was shown in the heatmap (**Figure 2D**). Additionally, the PRR gene expression levels amongst the three molecular subtypes are notably different, with cluster C being highly downregulated compared to the other two subtypes, according to the heatmap data. Three unique PRR molecular subtypes were confirmed by PCA (**Figure 2E**). The three PRR molecular subtypes have different prognoses, as seen by the K-M curves, with the C cluster having a clear prognostic advantage (*p*<0.05; **Figure 2F**). In addition, clusters A and B displayed more muscular immune cell infiltration than clusters C (**Figure 2G**).

### 3.3 Functional enrichment analyses of the PPR genes

**Figure 3:**
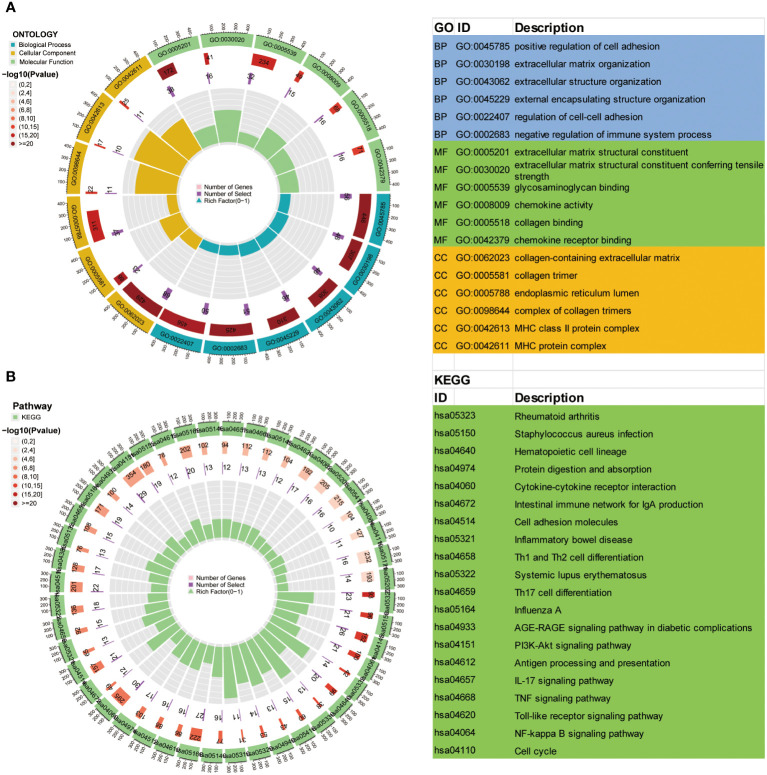
GO **(A)** and KEGG **(B)** analysis of DEGs between the three PRR molecular subtypes.

The intersection of DEGs between the different PRR clusters was generated (n=494, **Table S4**, **Figure S2A**) for functional enrichment analysis to explore the potential biological activities of the PRR molecular subtypes in BLCA. Using GO functional enrichment analysis (**Figure 3A**), we discovered that alterations in the TME, specifically in the regulation of the immune system process, extracellular matrix, and cell-cell adhesion, were the primary causes of the heterogeneity of PRR molecular subtypes. As a result of the KEGG analysis (**Figure 3B**), we discovered various biological processes and signaling pathways connected to molecular subtypes and BLCA. The PI3K-Akt pathway, the NF-B pathway, the Toll-like receptor pathway, and the TNF pathway are enriched in BLCA. In addition, we found that the intestinal immune network for IgA synthesis, cytokine-cytokine receptor connections, and cell adhesion molecules were all linked to the TIME infiltration. These findings provide insights into the mechanisms underlying platinum resistance and TIME in BLCA.

### 3.4 Comprehensive analysis of the platinum-resistant DEGs in BLCA

To further confirm the reliability of the PRR molecular subtypes, we conducted the clustering analysis on DEGs. The results were similar to the PRR molecular subtypes, identifying three gene-molecular subtypes named gene clusters A-C (**Figures S2B-D**). Similarly, the visualization of PCA suggested that the three molecular subtypes could be clearly distinguished based on the expression levels of DEGs (**Figures S2E**). Survival analysis showed significant differences in overall survival between the three clusters, with patients in cluster C having a significantly better prognosis than those in clusters A and B (**Figures S2F**). Next, we investigated the immune microenvironment of the three gene clusters. We found that the immune cell infiltration in gene cluster C was significantly lower than in the other two clusters (**Figure S2G**). All these results were similar to the findings of the PRR molecular subtypes.

### 3.5 Development of the PRR scoring system

**Figure 4:**
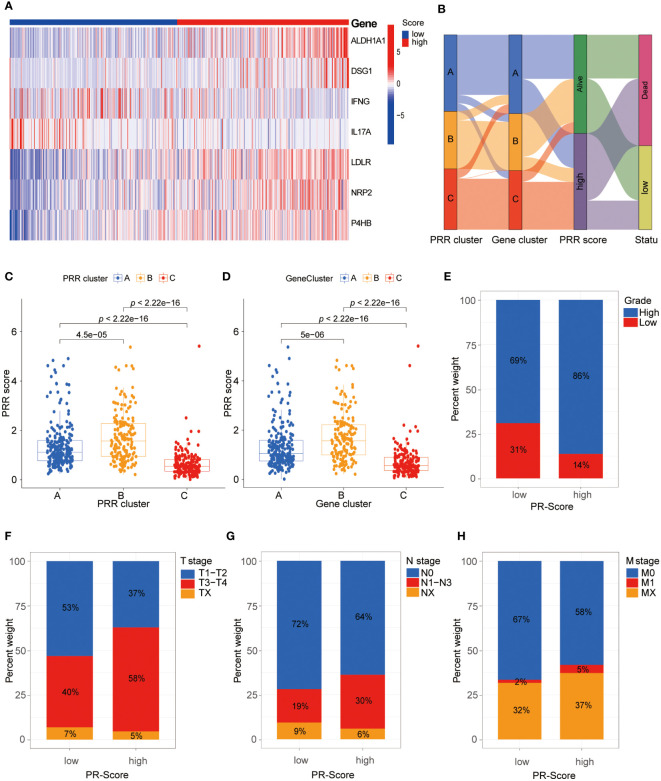
Comprehensive analysis of the PRR scoring system. **(A)** The heatmap visualizes the expression of the seven key PRR genes in the PRR scoring system. **(B)** The alluvial diagram reveals the relationship between the PRR clusters, gene clusters, PRR score, and survival status. **(C, D)** The relationship between the PRR scoring system with the PRR clusters and gene clusters. **(E-H)** The relationship between the PRR scoring system with the clinical-pathological features.

Based on the 264 PRR genes identified by univariate Cox proportional hazards regression analysis, we performed LASSO regression analysis and multivariate Cox proportional hazards regression analysis. Finally, we identified seven key genes to construct the PRR score system (**Figure S3**). The PRR score of each BLCA patient was calculated based on the following formula: PRR score= ALDH1A1*0.177833963780602 + DSG1*0.154877476230398 + IFNG*(-0.308143329982385) + IL17A*(-0.60024462463256) + LDLR*0.204175159362262 + NRP2*0.194069051932148 + P4HB*0.376758654397122. We classified BLCA patients into the high and low PRR score groups using the median value of 0.967. By combining the hazard ratios and expression heat map of 7 PRR genes, we found that the expression levels of genes with a hazard ratio of 1, such as aldehyde dehydrogenase 2C4 (ALDH1A), desmoglein 1 (DSG1), low-density lipoprotein receptor (LDLR), neuropilin 2(NRP2), and P4HB, were higher in the high-score group. In contrast, genes with a hazard ratio of 1, such as interferon-gamma (IFNG) and interleukin 17A (IL17A), were more highly expressed in the low-score group (**Figure 4A**). In addition, there was a significant correlation between the PRR molecular subtypes and the scoring system. Specifically, the PRR cluster C and the PRR gene cluster C were significantly associated with low scores, while A and B clusters demonstrated the opposite trend (**Figures 4C, D**). The alluvial diagram was utilized to effectively visualize the relationship between the PRR clusters, gene clusters, PRR score, and survival status (**Figure 4B**). In addition, barplots were used to visualize the relationship between the PRR score and the clinical-pathological features (**Figures 4E-H**). The results demonstrated that the high PRR score group was significantly associated with high grade, T3-4 stage, and N1 stage, all indicators of poor prognosis.

**Figure 5:**
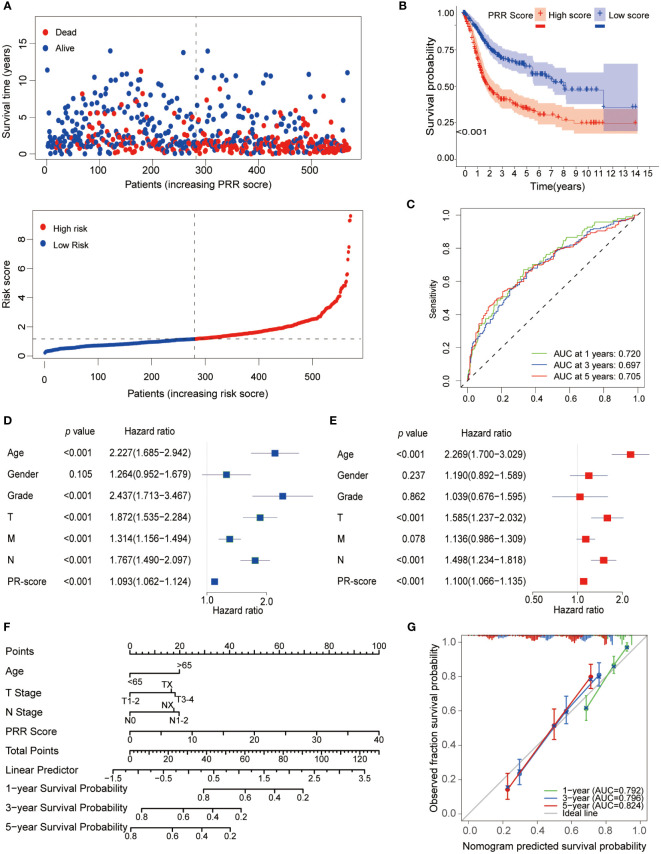
Prognostic analysis of the PRR scoring system. **(A)** The relationship between the PRR scoring system with survival time and status. **(B)** The K-M curve revealed that BLCA patients with a high PRR score had significantly worse survival than those with a low PRR score. **(C)** The ROCs demonstrated that the PRR score as a single predictor for patients 1, 3, and 5-year survival rates had good predictive performance. **(D)** Univariate and **(E)** Multivariate Cox regression analysis of BMR scores and clinicopathological characteristics. **(F)** The prognostic nomogram for predicting the 1-, 3- and 5-year overall survival of BLCA patients. **(G)** The calibration curves and ROCs are used for evaluating the predictive performance of the nomogram.

We then explored the relationship between the PRR score and prognosis. The survival analyses demonstrated that BLCA patients with a high PRR score had significantly worse survival than those with a low PRR score (**Figures 5A, B**). The PRR score as a single predictor for patients 1, 3, and 5-year survival rates exhibited ROCs of 0.720, 0.697, and 0.705, respectively (**Figure 5C**). Moreover, the univariate and multivariate Cox proportional hazards regression analyses revealed that age (HR=2.269, 95%CI=1.70−3.029, *p* < 0.001), T stage (HR=1.585, 95%CI=1.237−2.032, *p* < 0.001), N stage (HR=1.498, 95%CI=1.234−1.818, *p* < 0.001) and PRR score (HR=1.100, 95%CI=1.066−1.1351.0, *p* < 0.001) were the significant independent prognostic predictors for BLCA (**Figures 5D, E**). Subsequently, according to these independent prognostic factors, we constructed a prognostic model for BLCA (**Figure 5F**). The model has demonstrated more accuracy in predicting the 1, 3, and 5-year survival rates of BLCA than the single factor PRR score, with AUCs of 0.792, 0.796, and 0.824, respectively (**Figure 5G**). Furthermore, the calibration curves showed good agreement between the actual observation and the calculated probability (**Figure 5G**).

**Figure 6:**
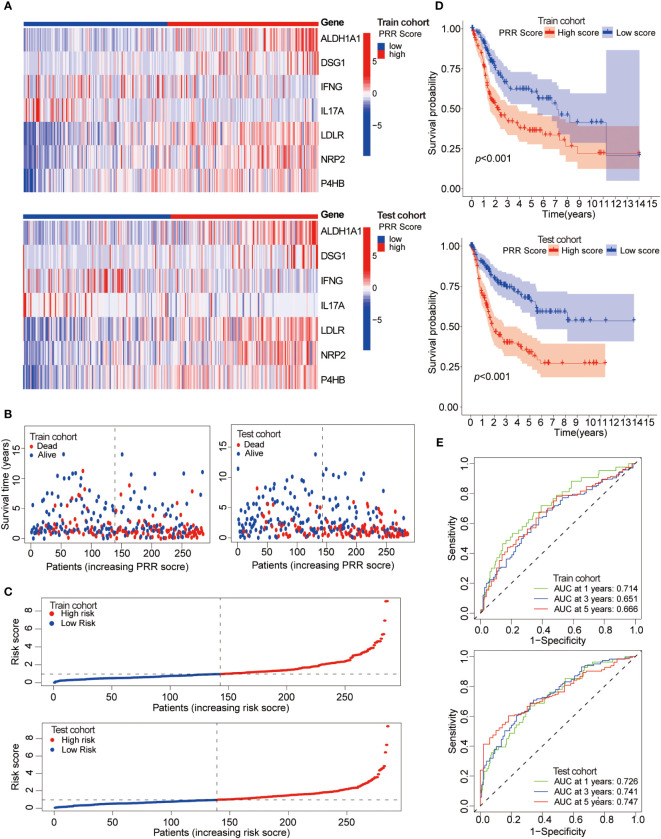
Assessment of the reliability and validity of the PRR scoring system. **(A)** The heatmap visualizes the expression of the seven key PRR genes in the training and testing cohorts. **(B)** The relationship between the PRR score, survival time, and status of BLCA patients in the training and testing cohorts. **(C)** The relationship between the PRR score and the survival status of BLCA patients in the training and testing cohorts. **(D)** The K-M cure for the different PRR score groups in the training and testing cohorts. **(E)** The ROCs of predicting 1, 3, and 5-year survival rates of BLCA patients in the training and testing cohorts.

Next, patients were randomly divided into training and testing cohorts in a 1:1 ratio to assess the reliability and validity of the PRR scoring system. It was found that the key PRR gene expression characteristics were consistent between the training and testing cohorts (**Figure 6A**). The negative correlation between the PRR score and survival status in BLCA patients was shown in both the training and testing cohorts (**Figures 6B, C**). The K-M survival curve indicated significant differences between groups. Higher mortality rates and shorter survival times were observed in all cohorts (**Figure 6D**). The AUC values for 1, 3, and 5-year survival were calculated and found to be high in all cohorts (**Figure 6E**).

All these results demonstrated that the PRR signature holds significant implications for characterizing BLCA. The prediction model based on the PRR score exhibits good reliability and stability, thereby providing a potential avenue for clinical implementation.

### 3.6 Relationship between the PRR scoring system and immune microenvironment

**Figure 7:**
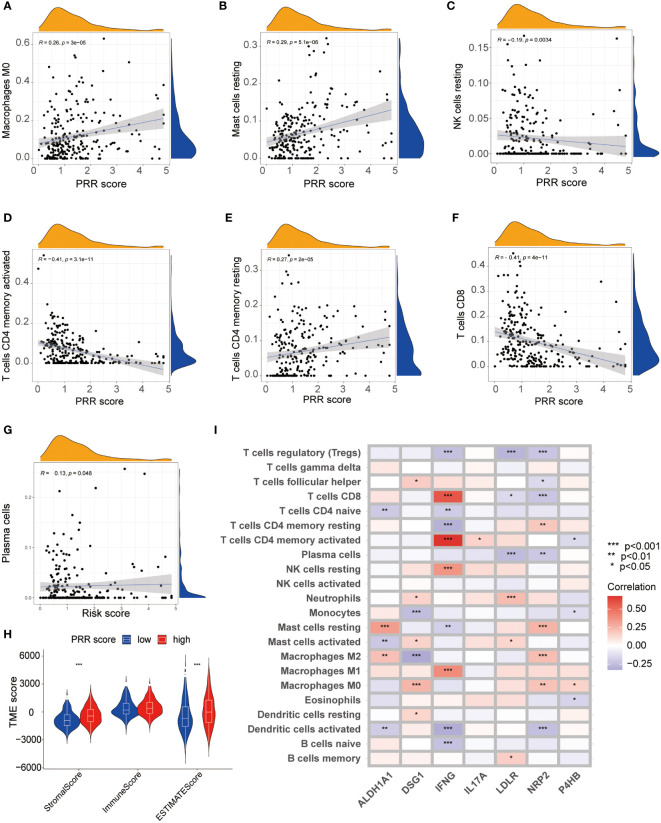
The relationship between the PRR scoring system and immune microenvironment. **(A-G)** The visualization of the relationship between immune cell infiltration and the PRR scoring system using CIBERSORT analysis (*p*<0.05). **(H)** Correlations between the PRR scoring system and Stromal Score, Immune score and ESTIMATE score. **(I)** The heatmap visualizing the correlations between the 7 key PRR genes and immune cell infiltration. (*p< 0.05; **p< 0.01; ***p< 0.001).

This study utilized multiple immune analyses to evaluate the association between the PRR scoring system and the TIME in BLCA. Firstly, we explored the correlations between the PRR score and infiltrating immune cells. The CIBERSORT analysis found a significant positive correlation between PRR score and M0 macrophages, mast cells resting, and cluster of differentiation 4+ (CD4+) memory T cells resting. In contrast, a significant negative correlation was observed between the PRR score and natural killer (NK) cells resting, CD4+ memory T cells activated, and luster of differentiation 8+ (CD8+) T cells (**Figures 7A-G**). The higher abundance of immunosuppressive cells in the high PRR score group suggested an immunosuppressive TME, consistent with a poor prognosis. The ESTIMATE score was calculated to evaluate the immune cell infiltration in tumor tissues. The result shows that the high RPP score group had a significantly increased ESTIMATE score compared to the low RPP score group, which is consistent with the results of CIBERSORT (**Figure 7H**). We generated a heatmap to investigate the relationship between the PRR signature and immune cells. Our results revealed a significant positive correlation between risk factors (ALDH1A1, DSG1, LDLR, NRP2, and P4HB) and M0 macrophages, M2 macrophages, resting mast cells, and neutrophils. In contrast, favorable factors (IFNG and IL17A) are significantly positively correlated with M1 macrophages, resting NK cells, activated CD4+ memory T cells, and CD8+ T cells (**Figure 7I**). We subsequently compared somatic mutations in the high and low PRR score groups and used waterfall plots to visualize the top 20 genes with the highest mutation frequency (**Figures S4A, B**). The results indicated no significant difference in tumor mutation burden between the groups. These findings provided important insights into the potential role of the PRR signatures in regulating the immune microenvironment, highlighting new avenues and directions for clinical treatment of BLCA patients with a risk of platinum resistance.

### 3.7 Relationship between the PRR scoring system and immunotherapy

**Figure 8:**
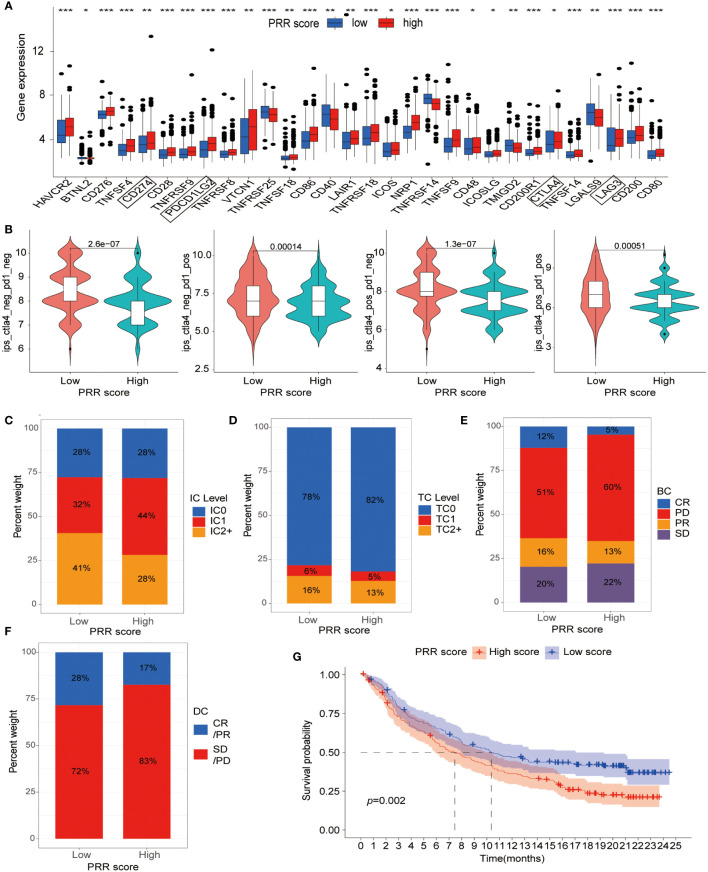
The relationship between the PRR scoring system and immunotherapy. **(A)** The expression of immune checkpoint-associated molecules in the low and high PRR score groups. **(B)** The efficiency of immunotherapy in BLCA patients with different expression of CLA-4 and PD1 by analysis of the TCIA dataset. **(C)** The IC and **(D)** TC levels of patients in the low and high PRR score groups. **(E, F)** The relationship between the PRR scoring system and treatment efficacy between the different score groups by using data from a clinical trial conducted by the IMvigor210 cohort. **(G)** The K-M curve analysis of the low and high PRR score groups in the IMvigor210 cohort. (*p< 0.05; **p< 0.01; ***p< 0.001).

We then shifted our focus to evaluating the association between the PRR scoring system and immune checkpoint expression levels. The results revealed a positive relationship between PRR scoring and most immune checkpoint-associated molecules, particularly the popular checkpoints CD274, programmed cell death 1 ligand 2 (PDCD1LG2), cytotoxic T-lymphocyte associated protein 4 (CTLA4), and lymphocyte activating 3 (LAG3), which exhibited significantly higher expression in the high PRR score group (**Figure 8A**). The upregulated expression of immune checkpoint-related molecules in the high-score group provided further evidence that the poor prognosis observed in these patients might be attributed to an immunosuppressive microenvironment. Subsequently, we explored the relationship between the PRR scoring system and immunotherapy in BLCA patients using data from the TCIA database. The results showed that regardless of the immune checkpoint molecules CTLA-4 and PD1 expression, the low PRR score group exhibited a much better response to immunotherapy than the high PRR score group (all *p*<0.001; **Figure 8B**). To further validate our results, we analyzed the data from a clinical trial conducted by the IMvigor210 cohort of metastatic urothelial cancer treated with anti-PD-1/PD-L1 therapy. Compared to the high PRR score group, the low PRR score group was associated with a higher proportion of immune cell (IC)2+ (41% *vs*. 28%), and a similar trend was observed for tumor cell (TC)2+ levels (16% *vs*. 13%) (**Figures 8C, D**), which once again confirmed the close relationship between the PRR signatures and the TIME. **Figures 8E, F** showed that the low PRR score group was significantly associated with a higher complete remissions/partial remissions (CR/PR) ratio (28% *vs*. 17%) and a lower stable disease/progressive disease (SD/PD) ratio (72% *vs*. 83%) compared with another group. In addition, the K-M curve analysis revealed that the low PRR score group had a better prognosis than the high PRR score group (*p*=0.002; **Figure 8G**). These results suggested that the PRR scoring system might have potential as a prognostic biomarker for BLCA patients with a risk of platinum resistance undergoing immunotherapy.

### 3.8 P4HB in BLCA progression and platinum resistance

**Figure 9:**
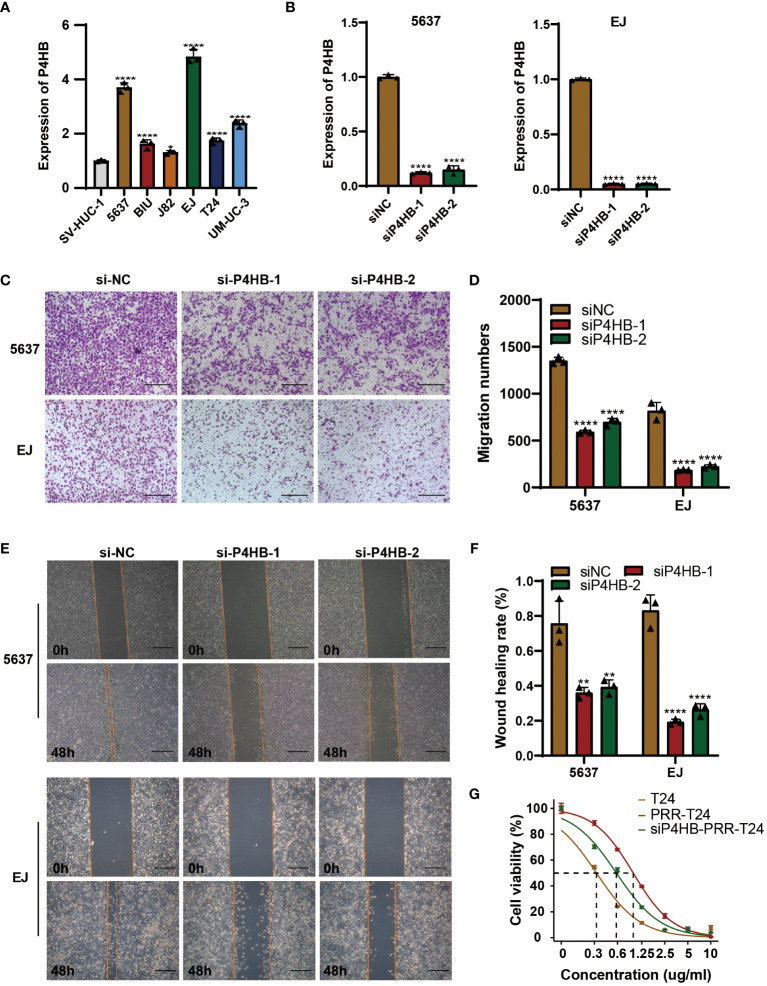
P4HB in BLCA Progression and Platinum Resistance. **(A)** Expression levels of P4HB in BLCA cell lines. qPCR analysis was performed to assess the expression levels of P4HB in various BLCA cell lines (5637, BIU, J82, EJ, T24 and UM-UC-3) and normal bladder epithelial cells (SV-HUC-1). **(B)** Knockdown efficiency of P4HB in 5637 and EJ cells. Representative images **(C)** and corresponding quantitative analysis **(D)** of the results were obtained from the wound healing assay, demonstrating the effects of P4HB knockdown in cell migration. Similarly, representative images **(E)** and corresponding quantitative analysis **(F)** of the results were acquired from the transwell assay, illustrating the impact of P4HB knockdown on cell migration. **(G)** CCK8 assay demonstrated the impact of P4HB knockdown on drug-sensitivity of platinum in BLCA cell. (*p< 0.05; **p< 0.01; ***p< 0.001; ****p< 0.0001).

To investigate the role of the PRR gene in cellular functions and its impact on the sensitivity of BLCA to platinum-based chemotherapy, we conducted a comparative relative expression analysis and prognosis assessment, ultimately selecting P4HB as the target for our study (**Figures S5A, B**). The expression levels of P4HB were significantly elevated in BLCA cell lines compared to normal bladder epithelial cells (SV-HUC-1) by qPCR experiment (**Figure 9A**). To investigate the role of P4HB in BLCA progression and platinum resistance, we established a siRNA transfection model for P4HB knockdown, and the inefficiencies were confirmed in 5637 and EJ cells by qPCR experiment (**Figure 9B**). The wound-healing and transwell assays showed that decreased P4HB expression in 5637 and EJ cells impaired cell migration ability (**Figure 9C**). Furthermore, P4HB knockdown in platinum-resistant T24 cells decreased the half maximal inhibitory concentration (IC50) to platinum-based chemotherapy drugs compared with platinum-resistant T24 cells by CCK8 assay (**Figure 9D**). These findings suggest that P4HB plays a crucial role in promoting BLCA progression and platinum resistance, highlighting its potential as a therapeutic target for personalized treatment approaches.

## 4 Discussion

Due to the biological and molecular genetic differences, BLCA patients may have different treatment responses and resistance outcomes to platinum-based chemotherapy (7, 9). The advent of molecular pathology diagnostic testing techniques has paved the way for a molecular-level approach to BLCA diagnosis and treatment. BLCA molecular subtypes are an important indicator of personalized tumor treatment, which holds immense clinical significance in prognosis and treatment selection (10). To investigate the relationship between platinum resistance and tumor heterogeneity in BLCA, the PRR genes were identified that related to prognosis. We classified BLCA into three PRR molecular subtypes, Cluster A, B, and C. Furthermore, we established a predictive risk model based on seven key PRR genes, which was found to be a strong independent prognostic tool closely associated with the immune inhibitory microenvironment. Our results may facilitate the development of precise immunotherapies for BLCA patients with a risk of platinum resistance.

In this study, we used a bioinformatics approach to identify the PRR signatures in BLCA. We performed consensus clustering, an unsupervised clustering analysis, to classify the tumors into three molecular subtypes based on their gene expression profiles (11). Cluster C had significantly downregulated expression of the PRR genes compared to the other two clusters. Furthermore, we used the CIBERSORT algorithm to analyze the immune cell infiltration in the three molecular subtypes. We found that cluster C had lower immune cell infiltration with a better prognosis. Therefore, we hypothesize that the heterogeneities of BLCA may be closely related to the TIME.

GO and KEGG analyses suggested that the TME is closely related to the regulation of cell adhesion, extracellular matrix organization, and negative regulation of immune system processes. Specifically, the positive regulation of cell adhesion and extracellular matrix organization may promote tumor growth, invasion, and drug resistance (12–15), while the negative regulation of immune system processes may inhibit immune surveillance and clearance of tumor cells (16). The extracellular matrix structural constituent and collagen binding may play important roles in maintaining the stability of the TME and promoting tumor progression (15). In addition, the KEGG analysis revealed several pathways potentially involved in tumor pathogenesis and progression, such as the PI3K-Akt signaling pathway, TNF signaling pathway, and Toll-like receptor signaling pathway (17–19). These findings better explain the molecular mechanisms underlying platinum resistance and tumor progression.

Considering the potential association between platinum resistance with the tumor heterogeneity and clinical outcomes of BLCA, we developed the PRR scoring system based on seven key genes associated with platinum resistance. More specifically, ALDH1A1, DSG1, LDLR, NRP2, and P4HB were considered risk factors, while IFNG and IL17A were favorable genes for platinum resistance, which is consistent with the current studies (20–23). As expected, cluster A exhibited the highest PRR score, while cluster C had the lowest PRR score, and cluster B showed a moderate PRR score. Furthermore, patients with a low PRR score demonstrated superior overall survival rates compared to those with a high PRR score. Following adjustment for confounding factors, the PRR score demonstrated potential as an independent prognostic biomarker in BLCA. The ROC analyses revealed that the PRR score exhibited a favorable ability to predict 1-, 3-, and 5-year overall survival rates with high accuracy. Therefore, the PRR scoring system may represent a robust prognostic tool for BLCA.

The alterations in the TIME have been demonstrated to play a crucial role in platinum resistance in various types of tumors (24–26). Tumor-associated macrophages (TAMs) are critical in tumor progression and chemotherapy resistance (27). The TME and stage affect the two different phenotypes of TAMs, M1 and M2 macrophages. While M1 macrophages can induce an inflammatory response to inhibit tumor growth, M2 macrophages suppress the immune response and promote tumor progression (28). T cells are the primary component of the antitumor adaptive immune response in the TME. Higher infiltration of CD8+ T cells is associated with better prognosis and treatment response (29). CD8+ cytotoxic T cells (CTLs) can secret cytotoxic enzymes, including perforin and granzyme, exerting tumor-killing mechanisms (30, 31). The expression of inhibitory receptors such as PD-1 and CTLA-4 is related to the severity of dysfunctional T cell phenotype. If the tumor cells activate immune checkpoint signaling pathways (e.g., CTLA-4 and PD-1), they can escape from CTLs attacks (28). CD4+ T cells can promote the proliferation of CTLs, increase antigen presentation by dendritic cell (DC), facilitate CTLs activation, and promote the formation of memory CTLs (32). NK cells are a critical component of the innate immune system and are highly effective in recognizing and eliminating undifferentiated or poorly differentiated tumor cells (33). During tumor development, tumor-associated inflammation induces the accumulation of neutrophils in the TME. In the later stages, these neutrophils undergo a polarization switch to N2 neutrophils and significantly infiltrate the tumor tissue, thereby promoting tumor growth and progression (34, 35). Our study observed a significant negative correlation between the PRR score and the abundance of resting NK cells, activated CD4+ memory T cells, and CD8+ T cells. Furthermore, the risk genes associated with platinum resistance exhibited significant positive correlations with immunosuppressive cells, such as M0 and M2 macrophages, resting mast cells, and neutrophils. Conversely, the favorable genes were significantly positively correlated with M1 macrophages, resting NK cells, activated CD4+ memory T cells, and CD8+ T cells. These findings supported the important role of tumor-associated immune cells in BLCA.

Based on the high expression of immune checkpoint-related molecules in the high PRR score group, we hypothesized that there might be a correlation between the PRR score group and immunotherapy efficacy. We conducted analyses of the relationship between the PRR scoring system and treatment efficacy using data from a clinical trial conducted by the IMvigor210 cohort of metastatic urothelial cancer treated with anti-PD-1/PD-L1 therapy. The high PRR score group had a higher expression of immune checkpoint-related molecules, such as CD274, PDCD1LG2, CTLA4, and LAG3, which are known to be upregulated in the immunosuppressive microenvironment and dysfunctional immune cells and are associated with poor prognosis (36, 37). Patients with high PRR scores had a lower CR/PR ratio (17% *vs*. 28%) and shorter overall survival than those with low PRR scores. Although the high PRR score group had higher expression of the immune checkpoints, the significant suppression of CD8+ cells enabled the tumor cells to escape from immune cell attack, which confirms the significantly reduced effectiveness of immunotherapy and poor prognosis in BLCA patients with a high PRR score.

Our findings revealed significantly elevated expression levels of P4HB in BLCA cell lines compared to normal bladder epithelial cells, suggesting its potential involvement in BLCA development. The recent meta-analysis containing the data of 4121 cancer patients by Wang, F. et al. demonstrated that high P4HB expression was significantly correlated with shorter OS (HR = 0.681; 95% CI, 0.560–0.802; P < 0.001) (38). The siRNA-mediated knockdown of P4HB in 5637 and EJ cell lines resulted in a substantial decrease in their migration abilities, indicating the critical role of P4HB in promoting cell migration and predicting survival in BLCA.

Drug resistance is a significant obstacle to successful treatment of cancers. It has been reported that P4HB played an important role in modulating chemoresistance in liver cancer, BLCA, and malignant glioma (39–41). In our study, the enhanced sensitivity of T24 platinum-resistant BLCA cells to platinum-based chemotherapy drugs upon P4HB knockdown suggests that P4HB may contribute to platinum resistance in BLCA. Platinum-based chemotherapy is a widely used treatment for BLCA, but resistance to these drugs remains a significant clinical challenge. Our study suggests that targeting P4HB could potentially overcome platinum resistance and improve treatment outcomes for BLCA patients.

Our study has several limitations. Firstly, it was a retrospective study, and the results need to be validated in prospective studies. Secondly, the sample size of the IMvigor210 cohort was relatively small, and more clinical trials with larger sample sizes are needed to further evaluate the relationship between PRR score and the efficacy of immunotherapy. Thirdly, the mechanisms underlying the role of the PRR signatures in the development of BLCA need to be further investigated.

## 5 Conclusion

In conclusion, our study demonstrated that the PRR molecular subtypes and the scoring system were significantly associated with clinical-pathological features, the TIME, and prognostic features, highlighting their potential as clinical tools for personalized therapy for BLCA patients.

## Data availability statement

The datasets presented in this study can be found in online repositories. The names of the repository/repositories and accession number(s) can be found in the article/**Supplementary Material**.

## Ethics statement

Ethical approval was not required for our study due to the fact that our research exclusively involved publicly available, de-identified data from databases like TCGA and GEO. The studies were conducted in accordance with the local legislation and institutional requirements. Written informed consent for participation was not required from the participants or the participants’ legal guardians/next of kin in accordance with the national legislation and institutional requirements because as these datasets maintain strict privacy protocols, all patient information is anonymized, rendering individual patient consent and ethical approval unnecessary.

## Author contributions

SX: Validation, Writing – original draft, Writing – review & editing, Data curation, Project administration. SL: Conceptualization, Methodology, Software, Visualization, Writing – review & editing. JZ: Methodology, Resources, Funding acquisition, Software, Supervision, Visualization, Writing – review & editing. JN: Formal Analysis, Methodology, Supervision, Writing – review & editing. TL: Formal Analysis, Methodology, Supervision, Writing – review & editing. XL: Conceptualization, Methodology, Project administration, Supervision, Writing – review & editing. LC: Conceptualization, Methodology, Project administration, Supervision, Writing – review & editing. BF: Conceptualization, Methodology, Project administration. JD: Conceptualization, Methodology, Project administration, Resources, Supervision, Writing – review & editing. SHX: Conceptualization, Funding acquisition, Investigation, Methodology, Project administration, Resources, Supervision, Writing – review & editing.
