# Supplementary material for: Deciphering the immunological and prognostic features of bladder cancer through platinum-resistance-related genes analysis and identifying potential therapeutic target P4HB
Source: Front Immunol. 2023 Sep 18;14:1253586. doi: 10.3389/fimmu.2023.1253586 (PMC10544894; doi:10.3389/fimmu.2023.1253586)
Supplement: Supplementary file 6 [file DataSheet_6.docx]

**Supplement materials**

Table S1

The merged dataset of TCGA and GEO databases was obtained after removing the batch effect.

Table S2

The intersection genes between the dataset and the PRR genes.

Table S3

864 PRR genes were selected via univariate Cox proportional hazards regression analysis.

Table S4

The differential expression genes for the different PRR subtypes.

Table S5

The results of different expression genes analysis.

Table S6

Raw data of Bladder cancer in TCIA dataset.

Table S7

Raw data of Bladder cancer in IMvigor210 dataset.

Table S8

The PRR genes for the PRR scoring system.

Table S9

The results of three molecular subtypes, with 237 patients in Cluster A, 174 patients in Cluster B and 183 patients in Cluster C.

Table S10

882 differentially expressed genes (DEGs) through differential analysis to further investigate the potential biological functions of BMR-associated subtypes.


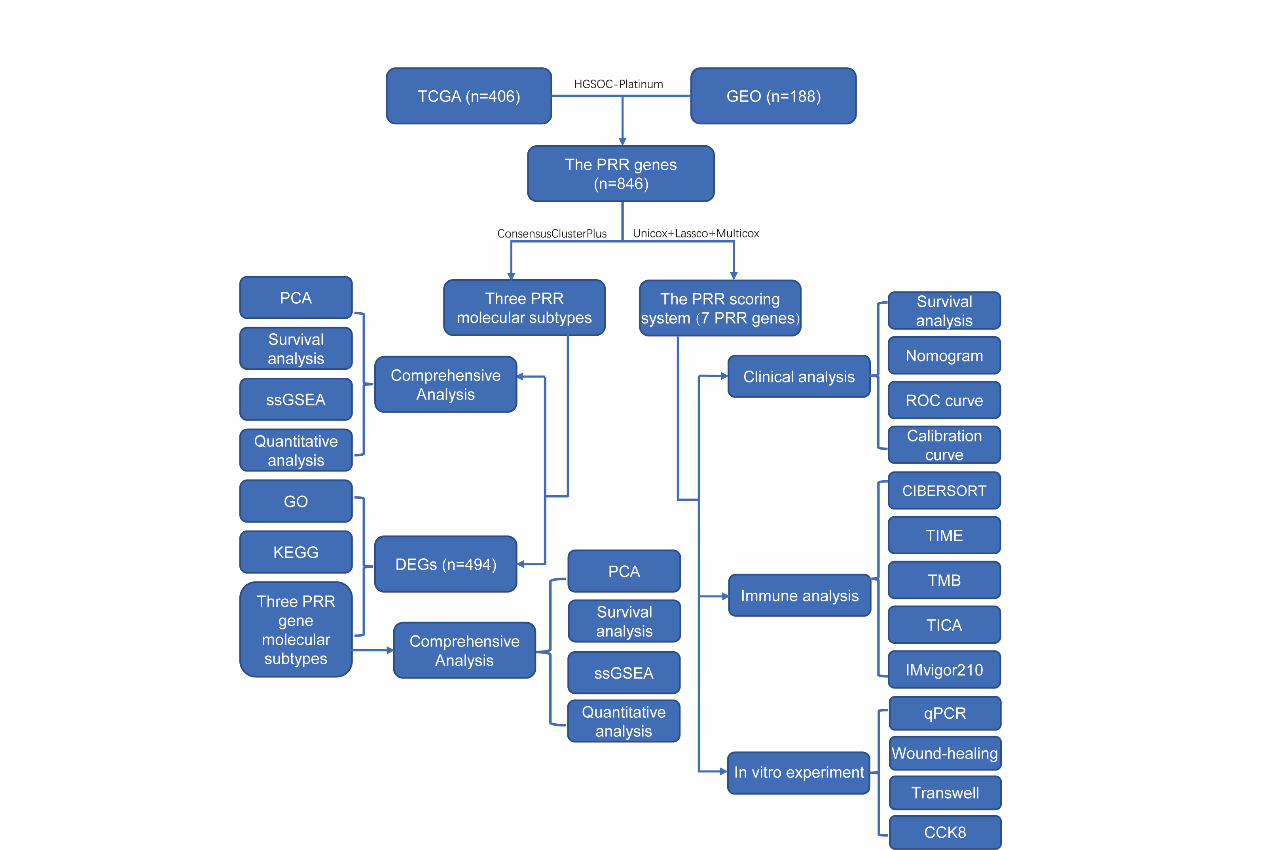


Figure S1. The flow diagram of this study.


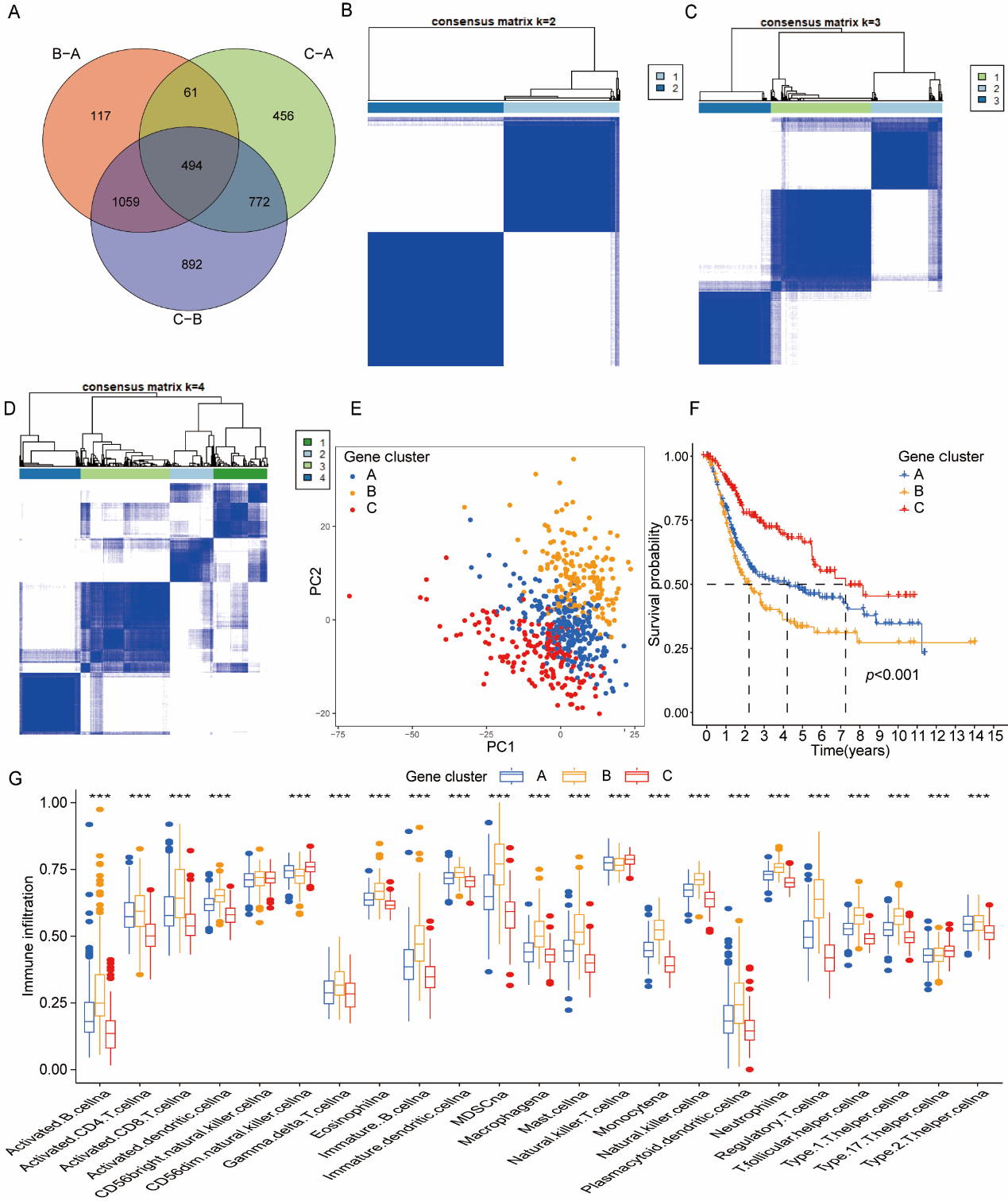


Figure S2. Identification of the PRR gene molecular subtypes in BLCA. (A) The intersection of differentially expressed genes (DEGs) between the different PRR clusters. (B-D) The consensus matrix heatmap demonstrated that the optimal clustering solution for consensus clustering was K=2. (E) PCA of the DEGs identified three different PRR gene molecular subtypes. (F) K-M curves for overall survival of the three gene clusters. (G) Immune cell infiltration abundance of the three PRR gene clusters. (**p*< 0.05; ***p*< 0.01; ****p*< 0.001)


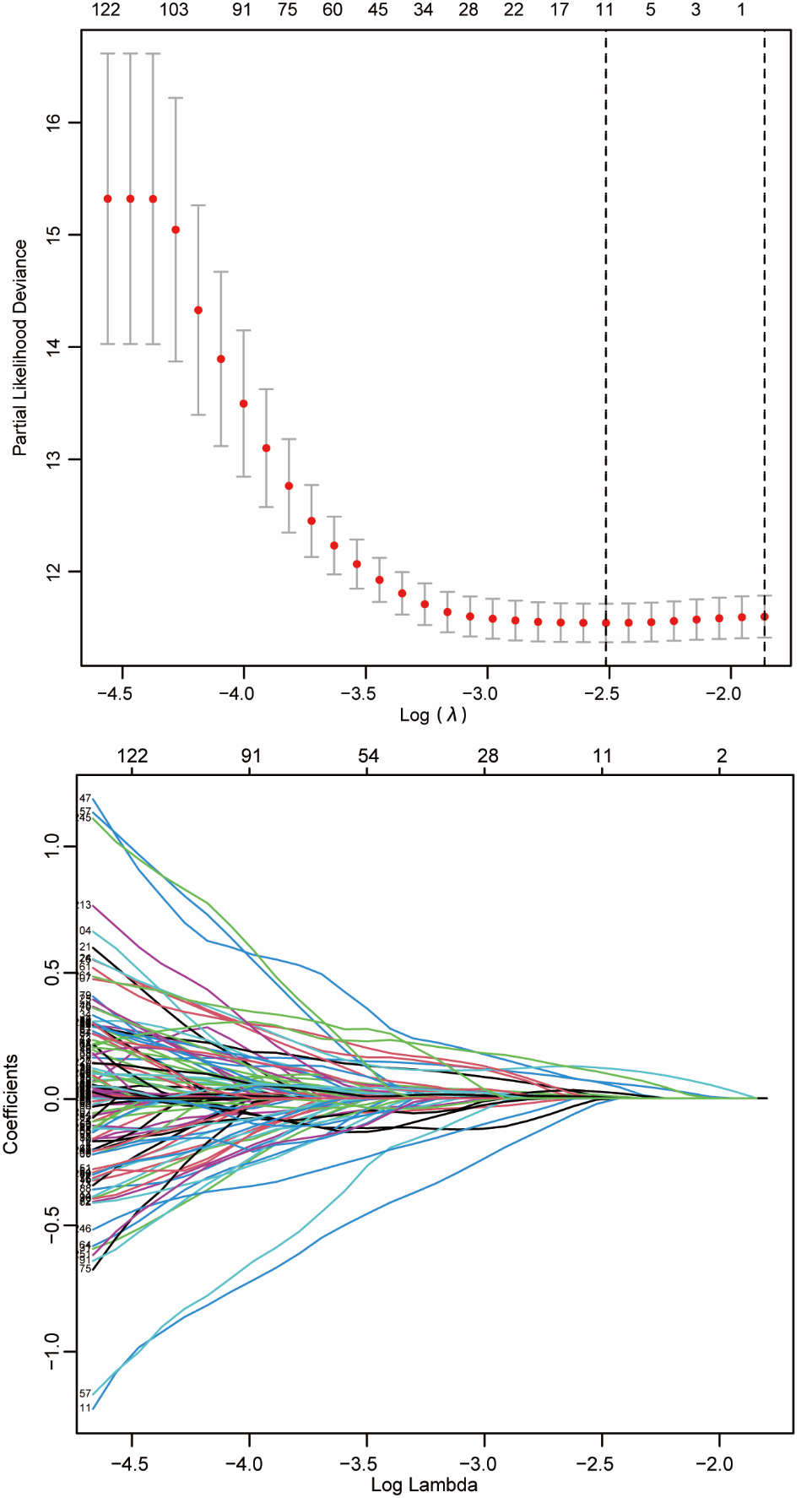


Figure S3. LASSO regression analysis to initial determine the key PRR genes for the PRR scoring system.


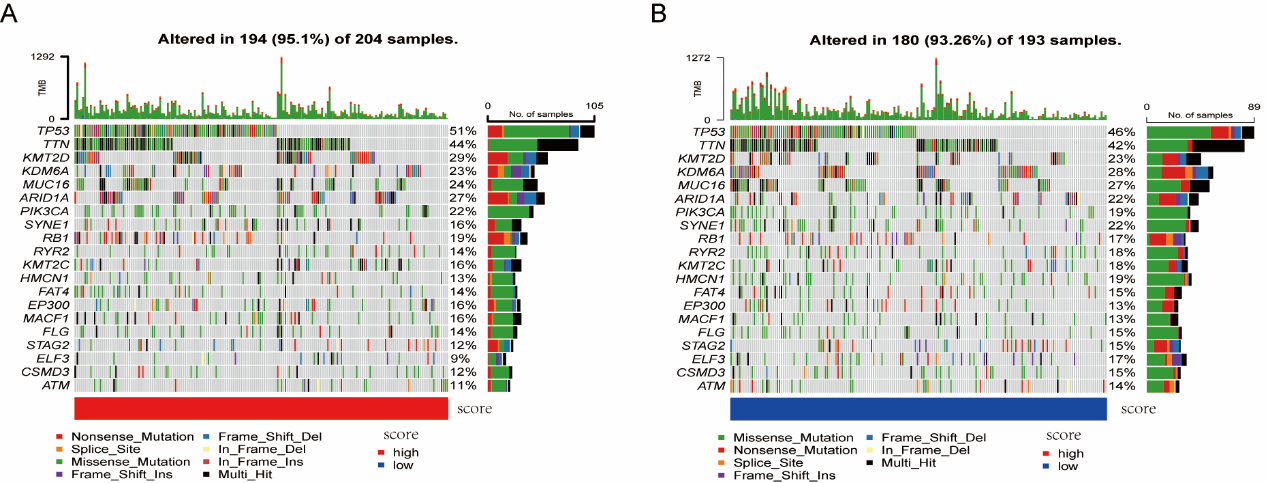


Figure S4. The relationship between the PRR scoring system and the somatic mutations. No significant difference in tumor mutation burden between the low and high PRR score groups.


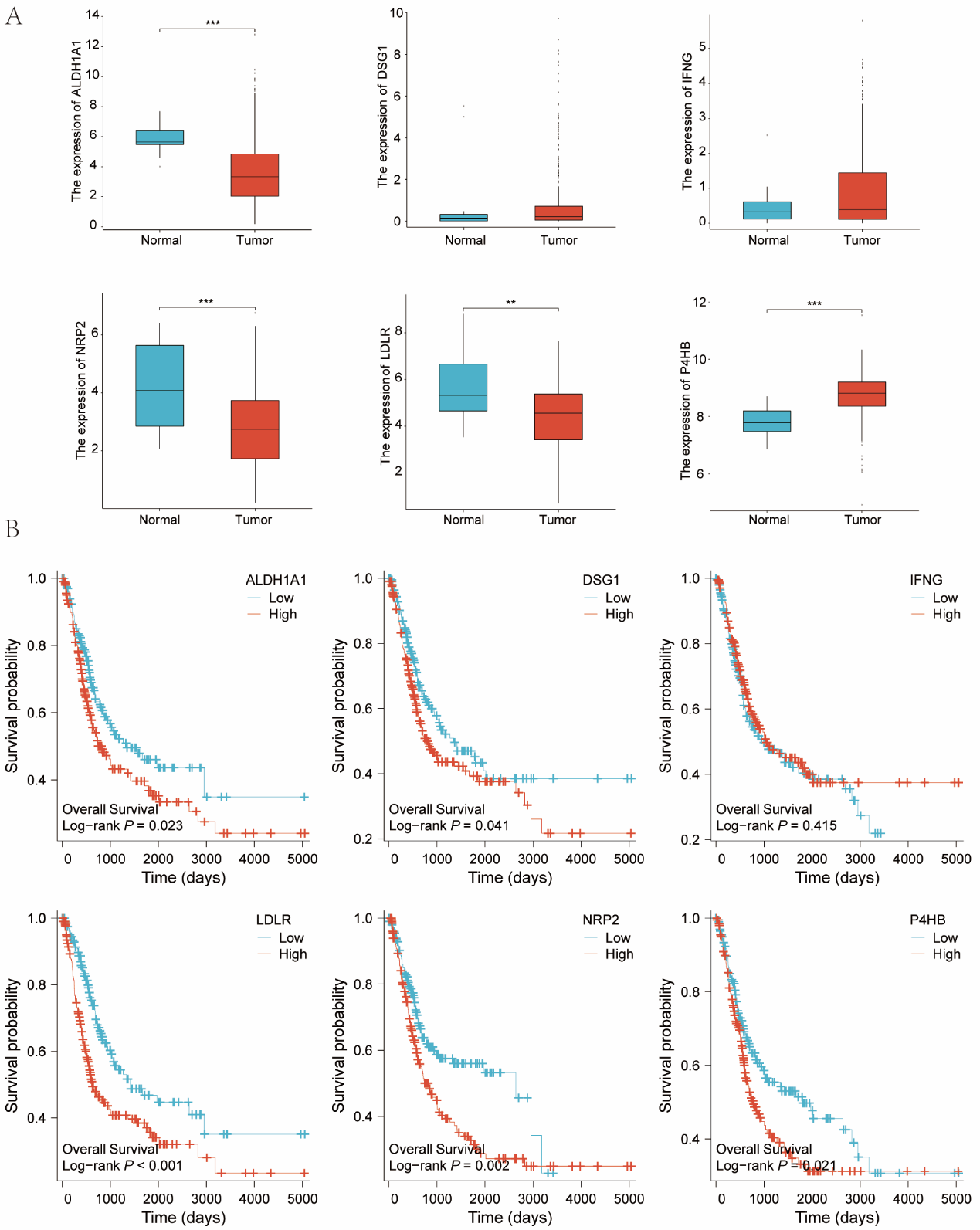


Figure S5. Comparative relative expression analysis (A) and prognosis assessment (B) among the key PRR genes.
