# Supplementary material for: Deciphering the immunological and prognostic features of bladder cancer through platinum-resistance-related genes analysis and identifying potential therapeutic target P4HB
Source: Front Immunol. 2023 Sep 18;14:1253586. doi: 10.3389/fimmu.2023.1253586 (PMC10544894; doi:10.3389/fimmu.2023.1253586)
Supplement: Supplementary file 7 [file Table_1.docx]

All R code, raw data and laboratory data can be download from this website：https://www.jianguoyun.com/p/DaRp2EcQl_2ACxjLqJAFIAA.

If you have any question about this, please feel free to contract me. E-mail: a382550906@163.com
